# From Host to Phage Metabolism: Hot Tales of Phage T4’s Takeover of *E. coli*

**DOI:** 10.3390/v10070387

**Published:** 2018-07-21

**Authors:** Elizabeth Kutter, Daniel Bryan, Georgia Ray, Erin Brewster, Bob Blasdel, Burton Guttman

**Affiliations:** 1 Bacteriophage Lab, The Evergreen State College, Olympia, WA 98505, USA; dwbryan@gmail.com (D.B.); geo.i.ray@gmail.com (G.R.); brewster.erin@gmail.com (E.B.); Robert.blasdel@kuleuven.be (B.B.); guttmanb@evergreen.edu (B.G.); 2 Department of Biosystems, KU Leuven, Leuven 3000, Belgium

**Keywords:** bacteriophage, Tevenvirinae, radiolabeling, genomic map, 2-D polyacrylamide gel electrophoresis, NEPHGE-SDS PAGE

## Abstract

The mechanisms by which bacteriophage T4 converts the metabolism of its *E. coli* host to one dedicated to progeny phage production was the subject of decades of intense research in many labs from the 1950s through the 1980s. Presently, a wide range of phages are starting to be used therapeutically and in many other applications, and also the range of phage sequence data available is skyrocketing. It is thus important to re-explore the extensive available data about the intricacies of the T4 infection process as summarized here, expand it to looking much more broadly at other genera of phages, and explore phage infections using newly-available modern techniques and a range of appropriate environmental conditions.

## 1. Introduction

The means by which bacteriophage T4 converts the metabolism of its *E. coli* host to one supporting phage production was the subject of decades of intense research, beginning in the 1950s. Indeed, no other large lytic phage’s infection process has been examined at T4’s level of detail, and results obtained with T-even phages provide much of the basis for our current interpretations and assumptions regarding lytic phage biology in general. Work with T4 and its relative T2 played key roles in the development of the science of molecular biology, from demonstrating that DNA is the genetic material, to the fact that viruses encode enzymes, to the existence and functioning of messenger RNA (mRNA), to the triplet nature of the genetic code [1]. The details of the T4 process are also in themselves highly relevant. About 200 of the 3000 unique tailed phages currently in GenBank are members of the Tevenvirinae subfamily, sharing most essential genes with T4, and hundreds of other such phages have been isolated, many of them for therapeutic purposes. T4-related phages are found in virtually every ecosystem. Much of the assignment of gene function in other kinds of phages is still based on data from T4, often without any independent verification. However, despite the decades of intense focus, some key aspects of T4’s infection process still remain mysterious.

A major tool in the early examinations of T4’s host-to-phage transition was radioactive labeling: using substrate molecules tagged with radioisotopes in order to precisely track the molecular changes within the cell. While biologists today have access to powerful next-generation tools like RNA-seq transcriptomics and modern metabolomics, radiolabeling still offers unique and powerful capabilities for exploring the phage infection process. Here, we review our radiolabeling studies of fundamental T4 biology over the last 55 years, hoping to encourage other labs to extend these techniques and areas of exploration to new concepts and to other genera of phages. This is especially important in light of the growing interest in phage therapy applications to help combat antibiotic resistance.

T4’s first alterations to the infected *E. coli* cell include inhibition of many of the host’s maintenance and constitutive functions. The host DNA is rapidly bound to the cell membrane throughout its length, and host DNA replication and transcription, translation of host mRNA, and cell division are all strongly inhibited within 1–3 min after infection. This thorough exploration of the T4 processes and of the experimental approaches that have led to this understanding is meant to provide a useful basis for the crucial task of exploring these processes in other phage-infected cells.

The limited other explorations of infection patterns across a variety of phage/host systems have revealed a much broader variation in those patterns than had been expected. For example, early data for *Bacillus* phage Φ29, based on radiolabeling techniques, first suggested that the T4 style of dominating host gene expression is not universal [2]. Recently, RNA-seq technology was used to explore the patterns of both host and phage transcription in *Pseudomonas aeruginosa* after infection by representatives of each of the seven genera of its professionally lytic phages [3,4]. In six of those phages, surprisingly much of the host transcription, as well as phage-mediated transcription of host genes, occurred during the infection process. Thus, in sharp contrast to T4, the host transcriptional machinery appears to have been left intact and able to let the bacterium respond to the specific stresses imposed by phage developmental processes as well as to at least some external stressors.

## 2. Genomic Map of T4

**Figure 1:**
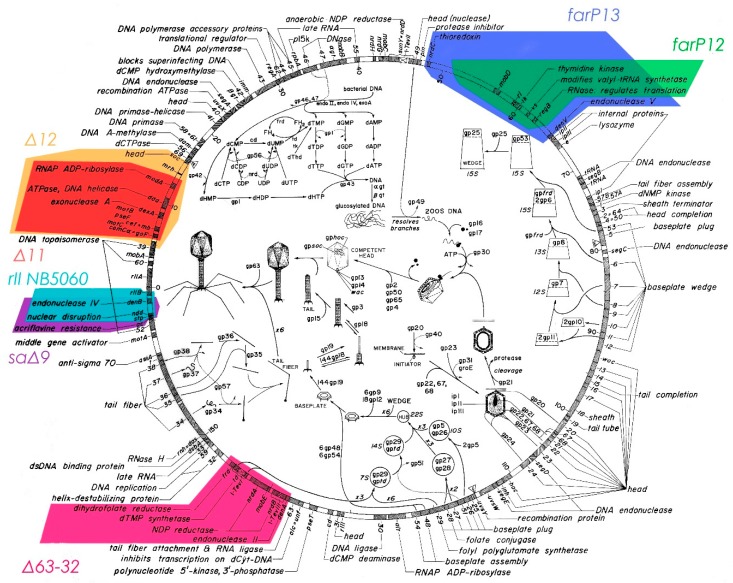
Genomic and functional map of bacteriophage T4: This map, tying various aspects of phage production to the relevant genes, is updated from the frontispiece by B. Guttman and E. Kutter for the 1994 ASM “Bacteriophage T4” book, in which the completed sequencing of the T4 genome was presented [5]. The mainly-structural late genes, beginning with gene *3*, are almost all transcribed in the clockwise direction on this map, while all of the genes expressed early and in the middle mode are transcribed in the counter-clockwise direction, including all of the genes in the big deletable regions that are indicated in color here.

The detailed T4 sequence-based map presented in Figure 1 was a major tool contributing to the ability to effectively use radioisotopes in studying the complexities of T4 infection and interpreting the resultant data [5]. Assembly of the sequence and production of this map depended on early work in the 1960’s, led by Bob Edgar and Dick Epstein. This included the isolation, characterization and mapping of conditional-lethal mutants which defined over 60 essential genes of T4 [6,7,8]; these are those genes classically identified by their gene numbers rather than by names in this map. Electron micrographs of mutant lysates allowed classification of many of those genes involved in encoding phage structures, while radioisotope-based enzyme assays facilitated identification of those genes responsible for various steps of nucleotide biosynthesis. In addition, radioisotopes, gel electrophoresis, and hand-read X-ray films were crucial to the early restriction mapping and sequencing of T4, as well as to the identification of its transcription control sites [9,10], complementing and refining the early recombination-based genomic mapping work [11].

The details of the T4 infection process are in themselves highly relevant, considering that T4-related phages (Tevenvirinae) are found in many different ecosystems, from the guts of all mammals, where they infect *E. coli* and *Shigella*, to the oceans, where they have been studied, targeting Cyanobacteria and *Acinetobacter*.

## 3. Initial Stages of T4 Infection

When T4 infects *E. coli*, it immediately begins transforming the host into a very efficient factory for phage production. The irreversible adsorption of the phage to its host is quickly followed by the adenosine triphosphate (ATP)-dependent transfer of the phage DNA into the cell (mechanism still unknown) and by the transcription and translation of over 100 small genes. A number of these small genes encode host-lethal products, as became clear during our early efforts to sequence those regions, when cloning of the DNA was still required. Many of these immediate-early proteins are encoded in the various deletable regions indicated in Figure 1, which were largely identified by Homyk and Weil [12]. The main purpose of most of these non-essential immediate-early gene products, which we called “monkey wrenches”, seems to be for adjusting the cell metabolism in a variety of ways that presumably make it more effective at phage production under at least some conditions [13]. However, the specific mechanism has been determined for only a few of them, despite extensive efforts in many labs, as shared informally at various early biennial Evergreen T4 phage meetings. Versions of most are found in a large fraction of the other T4-related phages that have been sequenced [14], appearing to indicate their broad usefulness under some sort of conditions.

## 4. 2-D Polyacrylamide Gel Electrophoresis Studies of T4 Proteins

**Figure 2:**
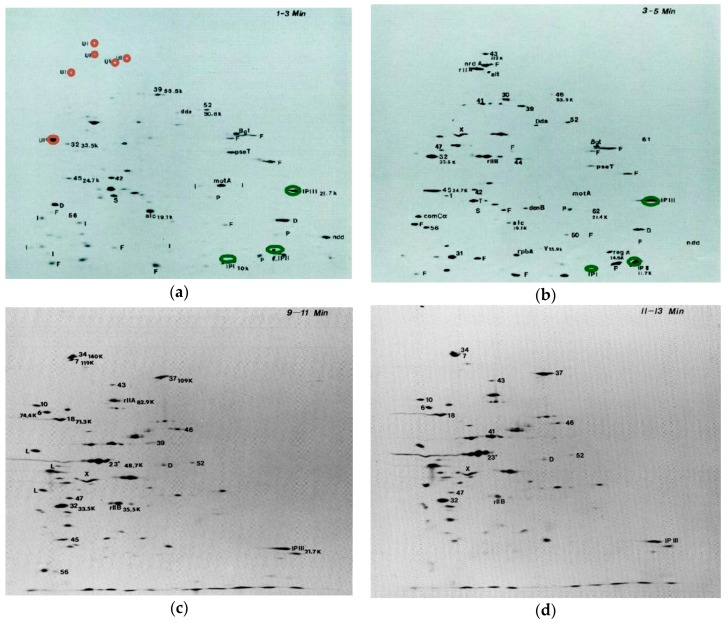
Two dimensional (2D) polyacrylamide gels of protein expression at 1–3 min (**a**); 3–5 min (**b**); 9–11 min (**c**), and 11–13 min (**d**) after T4 infection: An exponential-phase culture of *E. coli* B in M9 at 37 °C was infected with T4D at an MOI of 10. At the indicated times, samples were transferred to small flasks containing 14C mixed amino acids. Two minutes later in each case, a 3-min chase of unlabeled amino acids was administered and the cells were chilled, collected by centrifugation, lysed by boiling, and analyzed by two dimensional electrophoresis with nonequilibrium pH gradient electrophoresis as the first dimension, and sodium dodecyl sulfate-polyacrylamide gel electrophoresis as the second dimension [5]. Labels on gels: UI: residual uninfected *E. coli* gene products; D: missing in T4 (*39–56*)Δ12 deletion; F: missing in T4 ΔFar P13 deletion; P: missing in T4 *PseT* Δ3 deletion; S: missing in T4 SaΔ9 deletion; I: other immediate early gene products; L: late gene product. (The full time-course set of gels is available, with further technical details, in [18] from which this figure has been adapted).

Pulse labeling with ^14^C- or ^35^S-labeled amino acids can effectively show exactly what proteins are being made during any given specific period of time during phage infection, particularly when coupled with the two dimensional (2D) polyacrylamide gel electrophoresis technique developed by O’Farrell [15]. This technique was soon found to be particularly effective for exploring early patterns of protein synthesis after T4 infection of *E. coli*, and for identifying many of the proteins encoded by the essential T4 genes, as identified by using amber mutants, and the various deletion mutants indicated in Figure 1 [16,17]. It showed that virtually all synthesis of host-encoded proteins ends within 1 min after infection of *E. coli* B by T4 at 37 °C (Figure 2a; [18]); the only host proteins labeled in the first 1–3 min are the four very large proteins marked in red in the upper left quadrant of Figure 2a. This complements the early work reviewed by Wiberg and Karam [19], indicating that T4 infection very quickly blocks ribosomal association with *E. coli* mRNA. The mechanism of this preferential exclusion of host mRNA remains a particularly interesting area for potential study. This system may involve a small difference in the spacing of the Shine-Dalgarno sequence between T4 and *E. coli* mRNAs, as well as one or more of about nine small ribosome-binding proteins observed by John Wiberg. Unfortunately, those studies were interrupted and the proteins lost without being characterized when Wiberg tragically succumbed to rapidly-developing Alzheimer’s, and to our knowledge, no one has yet followed up on this work.

**Figure 3:**
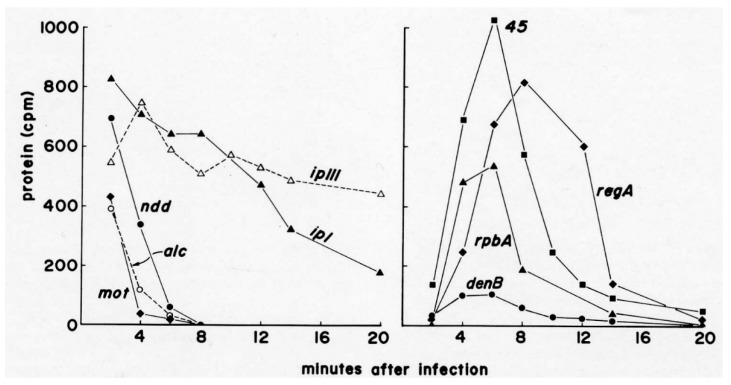
Quantitation of timing and extent of synthesis of various 14C-labeled T4 proteins: Approximate quantification of selected T4 proteins synthesized after infection. The Figure 2 gels were carefully aligned with the autoradiographs, spots for well-separated phage proteins were cut out and counted in a scintillation counter, and the values were corrected for protein size and acrylamide quenching. The actual number of molecules made each period was estimated by integrating the area under each curve and calculating ratios based on estimates in the literature. Koerner et al. reported that T4 makes about 4000 molecules per cell of ndd, used to bind the host genome to the cell wall [20]. Burke et al. estimated that 10,200 molecules/cell were made of gp45, involved in both DNA replication and late transcription [21]. Figure reproduced from [18].

The use of ^14^C-labeled mixed amino acids instead of ^35^S-labeled methionine and cysteine allowed the gels to show the precise ratios of all of the different proteins; this provided a level of detail which made it worth using an isotope that took a month or more to expose the films. For those proteins that weren’t too closely spaced on the gels, the actual ratios of proteins could be determined for given experiments by using the autoradiograms as a template to cut out the precise gel areas for counting in a scintillation counter (Figure 3). These counts are normalized for protein size and differences of quenching due to acrylamide concentration, thus assigning a quantitative absolute value to the ratios between amounts of different proteins, independent of external factors.

**Figure 4:**
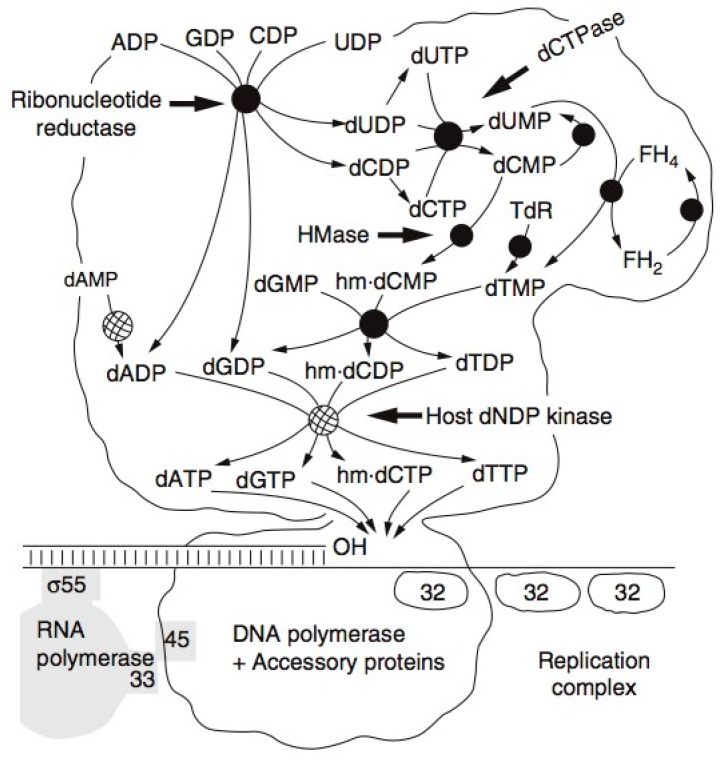
T4 DNA Replication Complex: The tight T4 complex of the enzymes responsible for nucleotide biosynthesis, DNA replication and late gene transcription. As host transcription is shut off, most of the ribonucleoside diphosphates are quickly channeled into producing a set of DNA precursor pools, in the process making hydroxymethyl deoxycytidine triphosphate (HMdCTP) rather than deoxycytidine triphosphate (dCTP). They flow in tightly linked fashion through the complex to the DNA polymerase, sustaining T4’s extremely rapid and efficient DNA replication.

One particularly surprising finding involves three rather small (8.5–20.4 kDa) highly basic proteins—Internal Proteins I, II and III (IPI, IPII, IPIII) marked in green in the 1–3 min gel—which are packaged in the phage head in thousands of copies each and injected into the host with the phage DNA. Surprisingly, they are actually made in large quantities early in infection (Figure 2 and Figure 4), as well as during late-mode expression when the phage structural proteins are made. This high-level early production suggests that these internal proteins may have some major early regulatory function in addition to their charge-balancing role in the packaging of the phage DNA in the capsid. Based on the timing of production, they might, for instance, bind to the ribosomes and aid in the rapid shut-off of host translation—a function for which no specific gene product has yet been identified, as mentioned above. Indeed, the many small host-lethal early phage genes have significant potential for serving as scaffolds for phage-inspired antimicrobials and bacterial modulators [22]. This would be worth exploring further with T4 as well, though earlier efforts in our lab and others were not successful.

## 5. T4 Nucleotide Synthesis and DNA Replication

T4 encodes almost all of the enzymes involved in the synthesis of its deoxyribonucleotides, and they have some unique properties. Radioisotopes played major roles in the identification by J.G. Flaks and S.S. Cohen of T4’s unique new enzyme, deoxycytidine monophosphate (dCMP) hydroxymethylase—the first virus-encoded enzyme discovered—as well as in much of the later work characterizing the whole set of T4 enzymes involved in nucleotide production [23]. These enzymes are mainly produced between 3 and 8 min after infection (Figure 3), along with the DNA polymerase and its complex of auxiliary proteins. They have the unique property of functioning together as a tight production-line complex (Figure 4) [24], rather than floating individually in the cytoplasm, and these complexes are in turn linked to the active DNA polymerase complex. A major unique feature of the complex is that it synthesizes dATP and deoxy-thymidine triphosphate (dTTP) in a 2:1 ratio to deoxyguanosine monophosphate (dGMP) and dCMP, reflecting the ratio in T4 DNA. This happens even when DNA synthesis is mutationally blocked, so this is not just the result of some sort of feedback mechanism. In sharp contrast, the four bases all occur in a 1:1 ratio in *E. coli* DNA, and no multienzyme complex is involved in their production in *E. coli*, or anywhere else that we know of beyond the T4-like phages. Most of the enzymes involved in the complex are T4-encoded, but the particularly abundant *E. coli* NDP kinase and deoxyadenosine monophosphate (dAMP) kinase are incorporated into the complex rather than T4 producing new enzymes of its own for those key steps [24].

## 6. Host DNA Degradation

**Figure 5:**
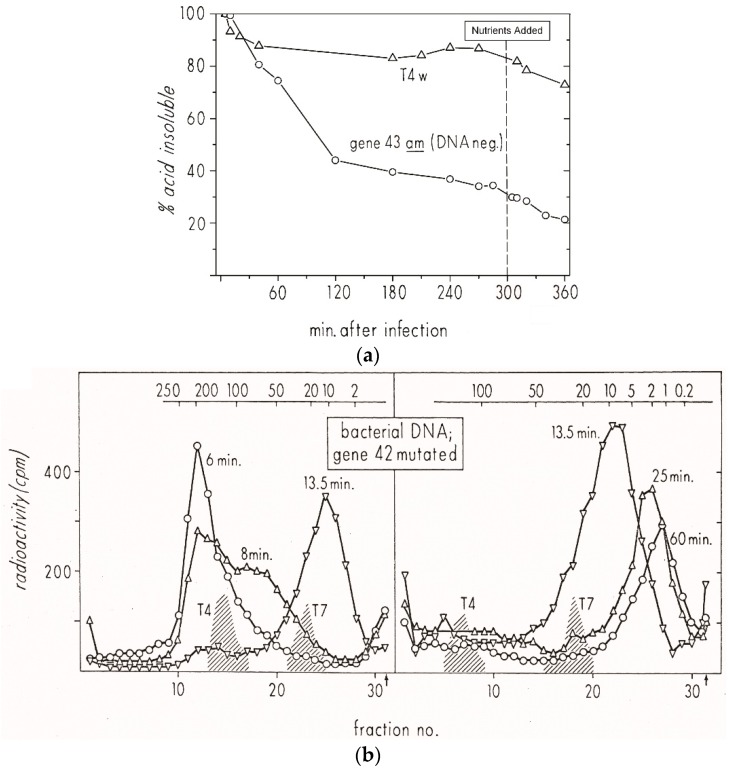

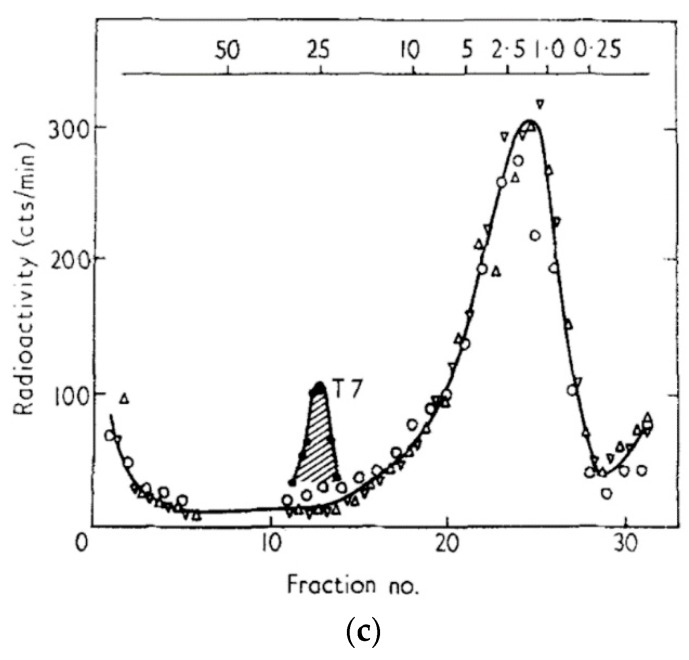
Host DNA breakdown and reincorporation into phage DNA during exponential phase T4 Infection. The top X axis of each figure indicates approximate molecular weight in megadaltons: (**a**) Status of ^3^HdT-labeled host DNA at various times during infection of *E. coli* B with T4D or T4 amN55x5 (dCMP HMase). The infection was carried out in exponential phase at 37 °C in glycerol-casamino acids (GCA) medium. Both T4 am4332 (DNA polymerase) and T4 amN55 (HMase) mutants produce no phage DNA and thus no phage when grown on a non-amber-suppressor strain such as *E. coli* B or ZK126; (**b**) Sucrose gradient analysis was used to determine the size of the acid-insoluble fraction of the host DNA at various times after exponential phase infection of *E. coli* B by T4D. The left panel shows fractions collected from samples lysed at 6, 8, and 13.5 min, the right panel shows fractions collected from samples lysed at 13.5, 25, and 60 min. Here, “T4” and “T7” refer to the phage DNAs used as sedimentation markers; (**c**) Size of the host DNA 25 min after infection with a T4 mutant defective in the gene 46 and 47 encoded exonuclease; here, all of the host DNA is only degraded to approximately the size of T7 DNA, and no host label is incorporated into the phage DNA which is being produced [25].

Initial studies of host DNA degradation after T4 infection, involving ^3^HdT labeling of the host genome, were carried out in 1966 as part of Kutter’s PhD thesis work on the transition from host to phage metabolism [25]. These studies took advantage of the fact that T4 uses hydroxymethylcytosine (HMdC) rather than cytosine in its DNA and makes a pair of endonucleases specific for cytosine-containing DNA to initiate the process of degrading host DNA while leaving the HMdC-containing phage DNA intact. They also made good use of T4 amber mutants that were available in, for example, gene *42* (dCMP HMase) or gene *43* (DNA polymerase). These blocked the synthesis of new phage DNA unless the infected host carried a gene encoding an “amber-suppressor” transfer RNA (tRNA); here, *E. coli* CR63, involved in the discovery of that amber-suppressor phenomenon, was used. Otherwise, the host DNA breakdown products were incorporated so efficiently into the new phage DNA that one scarcely saw any host DNA breakdown. This implies that the Figure 4 pathways of nucleotide metabolism also must include efficient entry points for the dNMPs generated by the gradual host DNA breakdown that occurs over the course of the infection cycle (Figure 5).

It quickly became obvious that studying T4’s breakdown of host DNA was only possible using mutants incapable of making phage DNA. Otherwise, the ^3^HdT released from the host DNA was very efficiently re-utilized in phage DNA (Figure 5a); this occurred even when a very large excess of non-radioactive dT was added to the medium during the process. However, most of that degradation, occurring mainly between 6 and 20 min after infection, was only observable using an *amber* mutant unable to produce phage DNA. In this case, a T4 mutant defective in HMase was used. HMase makes HMdCMP, which is then converted to HMdCTP for the phage DNA. The efficient phage dCTPase depletes the dCTP pool, preventing synthesis of cytosine-containing DNA. Similar results were observed with T4 DNA polymerase or dNMP kinase mutants.

Sucrose gradient analysis showed that the breakdown of the host DNA is a two-step process (Figure 5b); 25 min after infection, 20% of the host DNA is still acid-insoluble, in a single peak of about 3 million daltons. Further degradation depends on an exonuclease encoded by T4 genes 46 and 47 (Figure 5c), which is not specific for cytosine-containing DNA. It also plays a key role in T4 recombination. The initial step was eventually shown to involve a pair of cytosine-specific T4 enzymes: endonuclease II, encoded by T4 gene *denA*, nicks the DNA at very rare sites, while endonuclease IV, encoded by *denB*, cuts cytosine-containing stretches of the single-stranded DNA that is generated at those nicked sites [26].

## 7. Degradation of Host DNA during T4 Infection of Stationary-Phase *E. coli*

**Figure 6:**
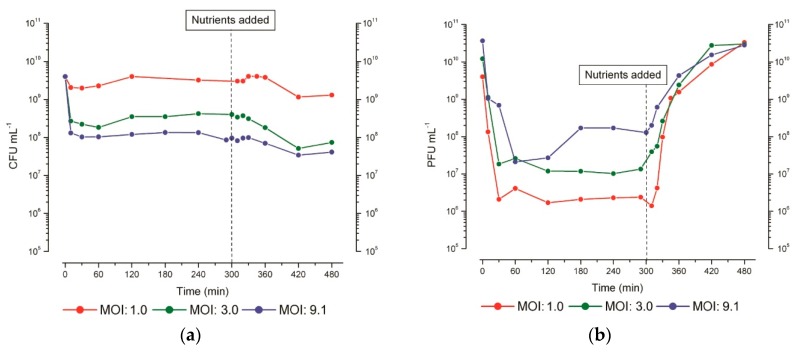
Stationary-phase phage production. Representative figures of (**a**) bacterial survivors (CFU) and (**b**) phage concentration (PFU) when T4 infection was carried out at three different multiplicities of infection (MOI) in parallel flasks split from the same 48 h-old culture of *E. coli* ZK126, with 0.12% *w*/*v* glucose and 0.1% *w*/*v* (CAA) re-added 5 h after infection. The addition could be delayed for at least 48 h. Figure reproduced from [27].

Far more recently, as a component of our investigation of T4 infection of stationary-phase *E. coli* [27], we used similar studies of the pattern of host DNA degradation to better understand the infection process when the host does not have the nutrients available to actively grow. When stationary-phase *E. coli* ZK126 (grown in M9 minimal medium for 48 or 72 h) was infected with T4, two general patterns of infection could be observed (Figure 6). The predominant pattern at relatively low MOI, which we have previously described as “hibernation mode” [27], is characterized by successful phage adsorption and bacterial killing that is followed by an extended period of senescence, where little or no phage production is observed in studies extending up to at least 24 h post-infection. However, if glucose and casamino acids (CAA) are re-added at any point, there is very rapid production of a large burst (up to 200 phage per cell) of progeny phage.

Especially at high MOI, a different infection pattern can be seen which we refer to as the “scavenger response”. It is characterized by successful phage adsorption and bacterial killing followed by a small, gradual burst of phage production (averaging about one phage per cell, produced 120–180 min after infection), but with no further stimulation of phage production after the addition of nutrients. The regulatory mechanisms which control the type of infection pattern the infected cells follow remain unclear. It has been observed, however, that the choice of pattern seems to be determined on a cell-by-cell basis. Infections where phage production patterns showing both types of responses are observed at intermediate MOIs (Figure 6b).

**Figure 7:**
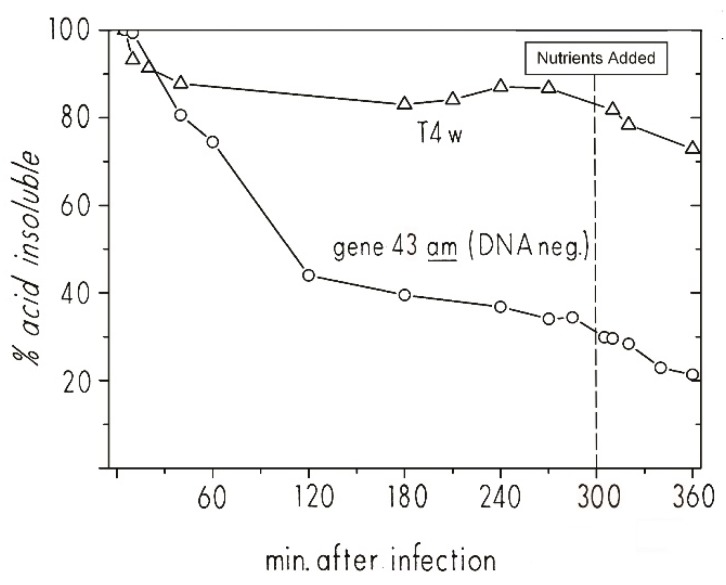
The breakdown and reutilization of host DNA and ^3^HdT DNA label reincorporation in stationary phase after T4 and T4 4332 amber DNA polymerase mutant infection: Host DNA degradation analysis of ZK126 that was labeled with tritiated thymidine during exponential growth and then infected at 48 h in stationary phase with either T4D or T4 am4332 (DNA polymerase). Figure reproduced from [27].

To more closely examine the infection dynamics of T4 “hibernation mode” in stationary-phase cells, we used ^3^HdT to pre-label host DNA during its exponential growth phase and then looked at the pattern of host DNA breakdown when phage infection was carried out at 48 h (Figure 7). Briefly, ^3^HdT and unlabeled dA were added early in the exponential phase of *E. coli* ZK126 growing in M9 minimal medium. At 48 h, it was split into two flasks and infected at an MOI of about 10 with T4D+ in one flask and T4 am4332 (carrying a DNA polymerase amber mutation) in the other. When infected with T4 am4332, 40% of the ^3^HdT became acid-soluble by 60 min post-infection, and 60% by 120 min after infection. The level continued to drop even after CAA and glucose were re-added at 3 hr. In the parallel T4D+ infection, there was a 10% reduction in ^3^HdT acid insolubility by 60 min, but almost no further reduction. Thus, though the process happens more slowly, the ^3^HdT-labeled host DNA patterns for both T4D and T4 am4332 infection of stationary-phase *E. coli* are similar to those observed in exponential phase infections.

The great depth of knowledge about T4’s infection process allow us to infer some specific characteristics of the “hibernation mode” infection process from given data. The DNA breakdown observed in the *amber* mutant infection indicates that the dC-specific T4 middle-mode endonucleases endoII and endoIV are produced fairly early in the course of stationary-phase infection, though substantially more slowly than in exponential phase. The lack of comparable net DNA acid solubilization in the wild-type infection indicates that the acid-soluble host nucleotides must have been rapidly reincorporated into T4 DNA, meaning that the genes responsible for T4 DNA replication were being expressed and, presumably, the replication complex was being formed. Endo II, endo IV, DNA polymerase, and the accessory proteins needed for T4 DNA synthesis are all encoded by genes expressed under middle-mode regulation. The lack of progeny phage production in the T4D+ infection over this time period (as enumerated by plaque-forming units) suggests a transcriptional or translational blockage of expression of the late-mode genes. This block is rapidly lifted by the addition of nutrients, allowing for the completion of progeny phage in much less time than T4 takes to go through its lytic cycle in exponential-phase cells. It remains to be determined whether that block is at the transcriptional or translational level. Of particular interest is our observation that the infected stationary-phase cell in hibernation mode remains viable enough to carry out efficient phage production upon nutrient addition for at least 48 h after the host chromosome has been extensively degraded, and host-directed regulation of metabolism is presumably blocked.

Before we carried out these radiolabeling studies, we were unable to determine the timing pattern of T4’s hibernation-mode infection process in stationary phase cells. It is very interesting that the pause occurs so late in infection, just before the amino acid-intensive production of large numbers of phage capsids. It is not yet clear whether late-gene transcription occurs coupled as usual to the DNA synthesis and/or is also delayed until nutrient addition, but the rapidity with which large numbers of phage are made after nutrient addition seems most consistent with at least some late-gene mRNA already being present. Also, late-gene transcription in exponential phase is directly linked to DNA replication, as indicated in Figure 4 and shown by the Geiduschek lab [28,29].

## 8. T4 Effects on *E. coli* Membrane Lipid Biosynthesis

Changes to lipid metabolism have been among the least studied aspects of the transition from host to phage metabolism. Some rather surprising effects have been observed in T4-infected *E. coli*, but their functions are still undetermined and little to no work has been published on other phage-host systems. In 1990, Dr. Kutter spent four months in Moscow, working with Vadim Mesyanzhinov on the T4 genome project and with scientists at the A. N. Bach Institute of Biochemistry on physiological studies. At the Bach Institute, the only spectrophotometer available to monitor bacterial growth was in the phospholipid lab of Volodja Eryomin, who soon inquired about the effects of T4 infection on lipid biosynthesis. Initially unaware of earlier studies in the field, they carried out a few initial experiments together that provided intriguing results, and Kutter invited Eryomin to visit the Evergreen phage lab to explore phage effects on lipid metabolism with us.

**Figure 8:**
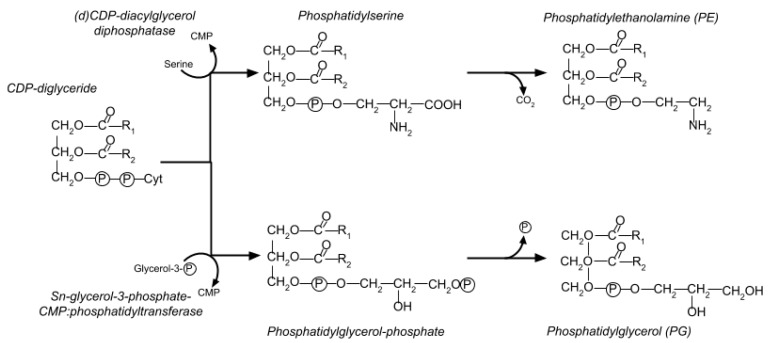
Biosynthesis pathway of PG and PE. Synthesis of both PG and PE begins by converting glycerol-3-phosphate to phosphatidic acid and thence into CDP-diglyceride, at which point the two pathways branch. For PE, CDP-diglyceride is converted into phosphatidyl serine and then into PE. The PG synthetic path converts the CDP-diglyceride into phosphatidylglycerol phosphate and then into PG.

*E. coli*’s membrane is composed of roughly 30% phospholipids. The majority are in the inner cytoplasmic membrane, which contains 67–75% of the cell’s total phospholipid content. Two major molecules are involved (Figure 8). Phosphatidylethanolamine (PE) makes up 70–80% of the total phospholipid, while the rest is phosphatidylglycerol (PG), sometimes in the form of cardiolipin (CL, or diphosphatidylglycerol) [30]. The biosynthesis pathway for PE and PG is shown in Figure 8.

**Figure 9:**
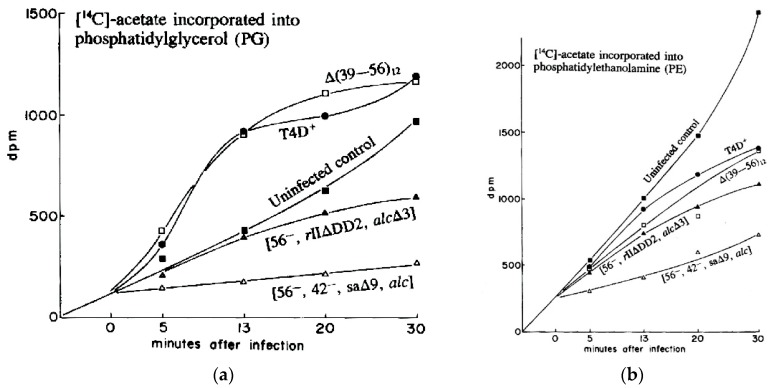
Kinetics of incorporation of [14C] acetate into (**a**) phosphatidyl glycerol and (**b**) phosphatidylethanolamine. Uninfected *E. coli* CR63 is compared with *E. coli* CR63 infected with T4D or with various multiple mutants of T4: [∆(*39–56*)12]; [sa∆9, *alc*∆3, am gene *42* (HMase), am gene *56* (dCTPase)], or [*rII*∆DD2, *alc*∆3, am dCTPase)]; *rII*∆DD2 partly overlaps sa∆9, as seen in Figure 1. Reproduced from [31].

**Figure 10:**
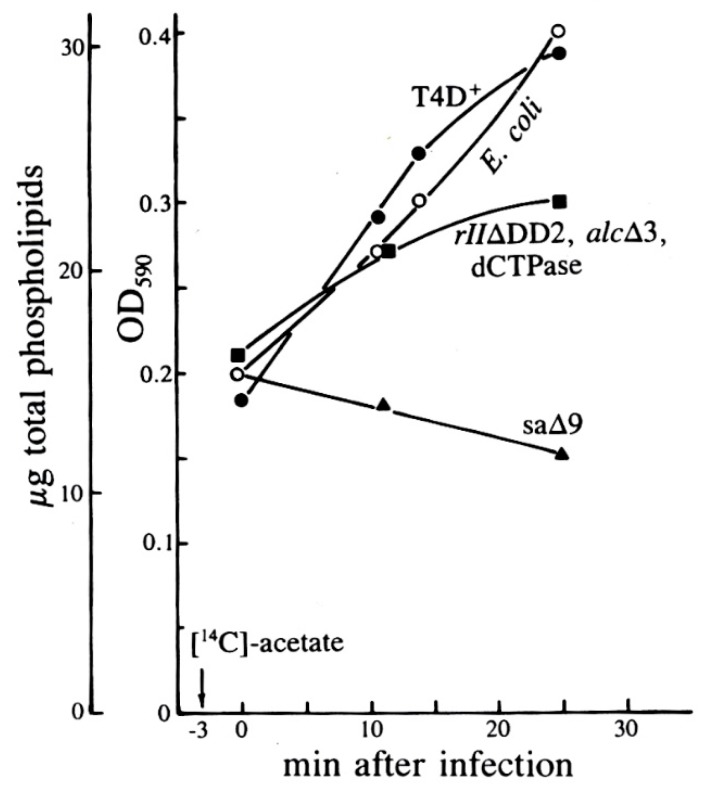
Total phospholipid levels after T4 infection of *E. coli* in exponential phase, as determined by the Victoria Blue R method of Eryomin and Poznyakov [32]. Uninfected cells are compared with cells infected with T4D, its mutant sa∆9, or the triple mutant (*rII*∆DD2, *alc*∆3, am *dCTPase*); the *rII*∆DD2 deletion partly overlaps sa∆9, as seen in Figure 1. Neither the dCTPase amber mutant nor the deletion in the *pseT/alc* region (see Figure 1) made any difference. Reproduced from [31].

In the Evergreen phage lab, we explored ^14^C-acetate incorporation into PG and PE as T4 infects *E. coli* K803, a K12 strain. To our surprise, while synthesis of PE gradually decreased after infection, synthesis of PG drastically increased over the first 13 min post-infection (Figure 9). As seen in Figure 10, this did not simply reflect an increased rate of turnover of PG, which would permit additional incorporation of the radioactive label, but a substantial net increase in the total amount of PG as measured chemically (Figure 10).

Studying the effects on PG and PE synthesis of the various multi-gene T4 deletion mutations depicted in Figure 1 proved fruitful (Figure 9 and Figure 10). When *E. coli* was infected by the T4 mutant sa∆9, which lacks about 3400 base pairs between genes *52* and *rIIB*, stimulation of all phospholipids was virtually abolished. This indicates that the post-infection lipid synthesis stimulation is genetically determined and therefore presumably adaptive in at least some situations. The deletion mutant *rII∆DD2*, which partly overlaps sa∆9, still allowed some lipid production, but only with comparable inhibition of both PG and PE production rates, and no stimulation of PG (Figure 9). Total phospholipid concentrations in sa∆9 infected cells confirm a reduction in all phospholipid synthesis over the course of infection, compared to infections with wild-type T4 and *rII*∆DD2, which both show a total increase in phospholipid concentrations (Figure 10).

This work shows that T4 has specific gene(s) for its effect on various lipid metabolism patterns, and thus that stimulation of lipid synthesis is not merely a passive consequence of infection or of phage release. Instead, there seems to be a specific adaptive mechanism that may eventually help determine the mechanism and role(s) of specifically continuing phospholipid synthesis, and actually stimulate phosphatidyl glycerol synthesis following T4 infection.

We later discovered that three separate teams had examined this phenomenon independently in 1968–1969, also using radiolabeling. However, it appears that little or no further such work was carried out until our 1990 studies, and we have found no other studies since [31]. John Cronan reported that *E. coli* B continued to incorporate 90% as much radiolabeled acetate into phospholipid after infection, and still cleaved preexisting phospholipids indiscriminately into non-membrane-bound fatty acids [33]. Furrow and Pizer were the first to observe that while bacterial synthesis of PE gradually stops after infection, the rate of synthesis of PG and cardiolipin actually increases, using ^32^P_i_ added at points during infection. They showed that the extent of incorporation of the labeled phosphorus into PG was reduced after 16 min post-infection, but PG:PE ratios remained higher than in uninfected cells. They concluded that the changes in phospholipid synthesis were due to a protein synthesized within 5 min after infection. The large farP13 and gene (*39–56*) T4 deletion mutants seen in Figure 1 were also tested but neither of these mutations altered the observed effects [34].

Using ^32^P-orthophosphate, Peterson and Buller observed the cessation of PE synthesis and stimulation of PG in *E. coli* K12 infected with T4, and in cells infected with ultraviolet (UV)-inactivated T4 or with empty T4 capsids, called “ghosts”, which are still able to adsorb to target bacteria and puncture the cell membrane [35]. They found that phospholipid synthesis slowed under those conditions, while PG synthesis was not stimulated; they speculated that the cessation of PE synthesis is not genetic, although PG synthesis possibly was. However, they searched in vain for deletion mutants that did not stimulate the PG synthesis. It was only later that the sa set of deletion mutants were isolated (on the basis of their sensitivity to acridine dyes, to which T4 is normally resistant). Our team happened to be using the above complex T4 mutant strains carrying either the sa∆9 or *rII*∆DD2 deletion because both eliminate T4’s cytosine-specific endonuclease IV, involved in initiating the degradation of host DNA, as we worked to determine the roles of substituting HMdC for C in T4 DNA—this has long been a major focus of ours.

In 1974, John Paul Merlie also used several radioisotopes to observe trends in lipid synthesis in T4-infected cells. In contrast to our studies, he found that incorporation of ^32^P into phospholipids was immediately inhibited upon T4 infection of *E. coli* K12 (in Tris medium at 37 °C) and that the effect was specific to PE. Synthesis of PG also was seen to decrease, though not as much, and the PG produced had a higher rate of turnover post-infection. While Merlie was unable to find a root cause, he ruled out that the difference between infected and uninfected cells involved sn-glycerol-3-phosphate-CMP:phosphatidyl transferase (the first step in PG synthesis after the PE/PG branch point). Merlie also observed that the inhibition event occurred before the formation of phosphatidylserine [36].

The T4 deletion mutant sa∆9 still produces viable phage at 50% the wild-type rate. This implies that neither the PG synthesis nor the products of any of the other genes in the deleted region are crucial for phage infection of standard lab strains of *E. coli*, and is in agreement with the findings of Nunn and Cronan that de novo phospholipid synthesis is valuable but not essential for T4 growth [37]. The genes missing from sa∆9 include: *52.1*, *ac* (acriflavine resistance), *stp* (host DNA restriction system inactivation), *ndd* (DNA-binding nuclear disruption protein), *ndd.1* to *ndd.6* (the products of which are unknown), and *denB* (endonuclease IV, which makes site-specific single-strand nicks in C-containing DNA and is needed for host DNA breakdown). The fact that the deletion of a relatively small region blocks the stimulation of phospholipid synthesis strongly suggests that the stimulation has some function in phage development and is not, for instance, just due to cell leakage or a general shut-down in metabolism. None of these genes are known to encode products that interfere with lipid synthesis or are related to proteins that do so, although several are theorized to be localized in the membrane based on their hydrophobic amino acid compositions [13]. The possible adaptive value of specific stimulation of PG is unclear [31]. It might conceivably be related to *E. coli*’s wide-ranging normal stress responses, since stress is known to alter the membrane phospholipid composition of uninfected *E. coli*. For example, cyanide [35], entering stationary phase [34], and benzyl alcohol [38] all cause PG:PE ratios in *E. coli* to rise. These may perhaps be measures to retain membrane integrity [38] or may simply be byproducts of changes in metabolism [35]. The cardiolipin:PE ratio also increases when the cell is osmotically stressed. PG is a precursor to cardiolipin, and in cells missing the cardiolipin synthase gene, the PG:PE ratio increases instead. The proportion of cardiolipin and PG also modulates ProP, an osmosensory transporter that modulates cell osmolality [39].

Alternatively, as Furrow and Pizer [35] speculated, this stimulation of PG could be a membrane-repair response. T4 creates a small hole in the peptidoglycan layer during infection, and infected cells are known to be more fragile for a short time after infection [31]. The lipid composition of the cell membrane is known to affect T4 assembly [40]. Parts of T4 tail and head morphogenesis are based in the membrane [41], and other T4 proteins, particularly several of those encoded in this region, are membrane-located.

PG stimulation could also still be a side effect of some other aspect of cell metabolism that is inhibited, or a byproduct of some other post-infection regulatory change. Peterson and Buller noted that the changes to lipid synthesis were concurrent with a reduction in host oxygen uptake [35]. For instance, (d)CDP-diacylglycerol, the branch point between PG and PE biosynthesis, may play a regulatory role in the cell. Phage mutants deficient only in the genes encoding dCTPase, EndoIV, and Alc do not show the specific stimulation of PG, but the fact that this lack of stimulation was also observed on a host that suppressed the amber dCTPase mutation suggests that dCTPase is not responsible for PG synthesis changes [31].

The use of radioactive labelling was crucial in identifying this unexpected effect of T4 infection on lipid biosynthesis. While several research teams have used radiolabelling to independently discover this fact, no modern research has used either this tool or any other line of inquiry to further explore this phenomenon. There remain several open questions: Which are the two or more genes that cause changes to phospholipid synthesis in T4-infected *E. coli*, and particularly that stimulate PG synthesis? What is the adaptive value for the bacteriophage of this stimulation? Does this property exist in other T4-related phages, and/or in other systems? Future studies could use radiolabelling and metabolomics to explore these possibilities and further our understanding of two fundamental model organisms.

## 9. Discussion

Though modern next-generation tools are immensely powerful and can provide amazing sensitivity, they are generally still very expensive and complex. They can also require very specific infection parameters that are difficult to achieve in all phage-host systems and metabolic contexts, such as simultaneous infection or a very low number of surviving bacteria post-infection. These methods also require a significant level of knowledge of the genomics of both phage and host to effectively generate and analyze the data produced. This makes the use of techniques such as transcriptomics and metabolomics especially challenging when exploring infections run under non-standard conditions, where achieving truly simultaneous infection and adequate killing of the host may be unattainable (as in T4 infections of *E. coli* in stationary phase.) By contrast, radiolabeling biological substrates allows for the direct and quantifiable observation of targeted biomarkers in ways that can also be tightly targeted to a given time period, require less specific experimental parameters, minimally impact the function of the labeled biomarkers, and require no prior genomic data to return interpretable results. Even as next-generation techniques continue to become more and more accessible, we believe that the older methods in our biological tool box, such as radioisotopic labeling, remain uniquely relevant and should be considered when possible. This is especially true in cases where only small amounts of low-energy isotopes are required, despite the unique challenges and regulatory requirements of working with radioisotopes. Ideally, such an approach should be coupled with the availability or isolation of amber mutants in at least some essential genes of new phages under consideration, to facilitate interpretation and associated testing of the results. It would also be very helpful to use radioactive labeling of DNA, protein and/or lipids to help clarify the meaning of previously observed results obtained with metagenomic and RNAseq techniques. One major factor we feel has been too little explored in other phage-host systems is whether or not the host DNA is degraded during the infection process. Evidence has suggested that lack of such degradation may play a key role in the recently-reported “superspreader” phenomenon, involving broad interspecies transmission of antibiotic resistance after infection of strains carrying such plasmids with some phages—Felix01-related phages in particular [42]. While T4 infection of an *E. coli* strain carrying an antibiotic-resistance plasmid led to very little transfer of the resistance plasmid when DNA released from the infection was transformed into a plasmid-free *E. coli* strain, infection with a T4 mutant strain (T4GT7) lacking the genes for making HMdC and for initiating degradation of the host DNA led to a very substantial increase in transformants. However, the number of transformants was still more than an order of magnitude less than the rate of transformation observed for the two Felix01-related phages in which the phenomenon has been identified to date.

Most of the work presented here was carried out largely by undergraduates at The Evergreen State College. The “Phagehunters” program initiated by Graham Hatfull has introduced tens of thousands of young people around the country to the phage, focusing largely on the isolation and sequencing of new *Mycobacterium smegmatis* phages, but now expanding to some other phage systems [43]. For relatively little money, parallel programs could be developed to support more physiological studies of phages of other bacteria, including *E. coli*, where a number of phages with genetic systems are already available. A major key area that has so far largely been neglected involves exploring phage infection under conditions more similar to those encountered in nature. Some of these conditions include anaerobically and/or in stationary phase. To date, there has only been limited work there, involving a few key *Pseudomonas* phages and T4-related Tevenvirinae. Such fundamental studies would be particularly useful and important in easily accessible phage-host systems where phages are being seriously considered for therapeutic applications, including *E. coli* and other enteric bacteria. Extensive further studies of various kinds of phages in conditions under which they might be used are clearly needed, and many that could be carried out in broad educational contexts could help inform therapeutic and biocontrol applications.
